# Corticosteroid-Associated Angiolipomatosis

**Published:** 2017-04-06

**Authors:** Eric Clayman, Kathryn King, Michael A. Harrington

**Affiliations:** Division of Plastic Surgery, Department of Surgery, University of South Florida Morsani College of Medicine, Tampa, Fla

**Keywords:** angiolipomatosis, angiolipoma, lipomatosis, lipoma, immunosuppression

## DESCRIPTION

**Figure 1:**
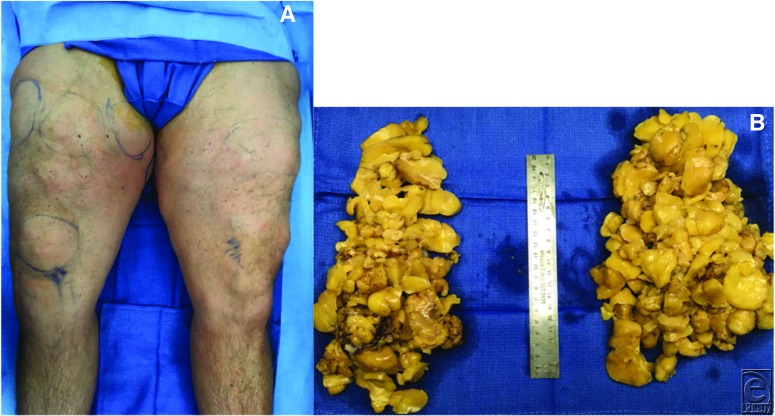
(A) Numerous subcutaneous nodules along bilateral lower extremities; (B) Gross pathology of six removed masses.

A 48-year-old man with a history of bilateral kidney transplant 33 years ago on long-term immunosuppression presented with 16 years of numerous subcutaneous masses on bilateral upper extremities and bilateral thighs (Fig 1a). He underwent excision of 6 of these masses, which pathology revealed as angiolipomas (Fig 1b).

## QUESTIONS

1. **What is an angiolipoma?**
2. **How do angiolipomas typically present?**
3. **What are the predisposing factors associated with the development of angiolipomatosis?**
4. **What is the preferred management of angiolipomatosis?**

## DISCUSSION

Angiolipomas are benign mesenchymal tumors composed of fat, capillaries, and stroma.^1^^,^^2^ These demonstrate a higher degree of vascularity on histologic examination as compared with lipomas, and histopathologic analysis is necessary to distinguish these two benign tumors. The vascular component of an angiolipoma varies from 15% to 50%, compared with less than 10% in lipoma.^3^ Lipomas are much more common than angiolipomas; less than 10% of specimens initially diagnosed as lipomas were angiolipomas after histopathologic analysis.^4^

Angiolipomas typically present as multiple, painful, subcutaneous masses ranging in size from 1 to 4 cm that initially occur following puberty and continue to occur over time. The most common location of lesions is the forearm but other locations include the legs and the trunk. These lesions occur in both men and women 20 to 30 years of age but more commonly in men.^1^ The lesions are nodular and well-circumscribed in appearance, have a rubber-like consistency, and, compared with lipomas, are more firm and often tender/painful.

The majority of angiolipomas are thought to occur sporadically; however, there are a minority of cases that have been associated with familial inheritance patterns and the use of certain medications.^1^^,^^4^^,^^5^ Indinavir, a protease inhibitor used in the treatment of HIV, is a medication that has been associated with the development of multiple angiolipomas.^5^ There are numerous documented cases in the literature associating long-term corticosteroid use with the development of numerous lipomas, most frequently occurring in an axial distribution—spinal epidural, retro-orbital, and mediastinal.^6^

Asymptomatic angiolipomas do not require medical or surgical treatment due to the benign nature of the tumor. However, they are often painful and, depending on their size and number, can be aesthetically unappealing and cause a change in behavior and lifestyle to conceal these subcutaneous masses. In addition, the pain associated with these lesions typically does not respond to analgesics.^7^ With regard to corticosteroid-induced lipomatosis, cessation or reduction of steroid therapy has not been shown to cause a consistent decrease or disappearance of lipoma sites.^6^ Surgical excision is currently the only documented treatment for removing angiolipomas. They are traditionally removed through single incisions but may require multiple incisions that can lead to significant disfigurement. Liposuction is a method that can be utilized without causing significant scarring, but poor visualization of the tumor and fragmentation of the tumor prior to pathologic diagnostic confirmation are potential drawbacks to this technique. Ronan and Broderick^8^ proposed a minimally invasive approach to removing numerous abdominal wall lipomas involving two 2- to 3-cm vertical midline incisions in the subxiphoid and supraumbilical areas and using a lighted breast retractor for visualization.

In this report, we have described a case of a 48-year-old man taking long-term immunosuppression secondary to a bilateral kidney transplant he received at 15 years of age. His immunosuppression regimen consisted of azathioprine and prednisone. He has no family history of multiple lipomatous masses. He described a sudden onset of development of these masses on his bilateral upper extremities and bilateral thighs at 32 years of age. Histopathologic evaluation of the masses removed was consistent with angiolipoma. The literature has shown numerous reports of long-term immunosuppression being associated with the development of lipomas in the spinal epidural space and mediastinum with the time to development following initiation of corticosteroid therapy ranging widely. The classic clinical presentation of sporadic angiolipoma is development of a few masses following puberty. Our patient began noting masses to his bilateral upper and lower extremities at 32 years of age, after approximately 16 years of immunosuppressive therapy. This case may represent an association between long-term immunosuppression and the development of angiolipomas.
